# Synthetic exposure with a CMOS camera for multiple exposure speckle imaging of blood flow

**DOI:** 10.1038/s41598-022-08647-6

**Published:** 2022-03-18

**Authors:** M. Chammas, F. Pain

**Affiliations:** Laboratoire Charles Fabry, Institut d’Optique Graduate School, CNRS, Université Paris-Saclay, 91127 Palaiseau, France

**Keywords:** Blood flow, Imaging and sensing

## Abstract

Speckle contrast imaging is an established technique to obtain relative blood flow maps over wide fields of view. A major improvement of the method relies on the acquisition of raw speckle images at different exposure times but requires simultaneous modulation of a laser pulse in duration and intensity and precise synchronization with a camera. This complex instrumentation has limited the use of multiple exposure speckle imaging. We evaluate here the use of a CMOS camera for a simplified approach based on synthetic exposure images created from the sum of successive frames acquired at a 1 ms exposure time. Both methods have been applied to evaluate controlled flows in micro-channels. The contribution of noises to the speckle contrast have been quantified and compared. Dark, readout and shot noise contributions to the total contrast remain constant for modulated exposure, while all these contributions decrease with increasing exposure time for synthetic exposure. The relative contribution of noises to speckle contrast depends on the level of illumination and the exposure time. Guidelines for flow measurements and limitations of the use of a CMOS camera with a limited frame rate for synthetic exposure acquisition scheme are discussed. The synthetic exposure method is simple to implement and should facilitate the translation of multiple exposure speckle imaging to clinical set-ups.

## Introduction

Speckle contrast imaging is an efficient tool to monitor in vivo relative blood flow changes and tissue perfusion. It has been used in several medical fields such as rheumatology, dermatology, ophthalmology, and neurology^1^. Multiple exposure speckle imaging (MESI) has proven to be more accurate than simple exposure laser speckle imaging for flow quantification due to its ability to differentiate the contribution of static scatterers (i.e. bones, cartilages) from that of the moving scatterers (i.e. red blood cells) to the speckle patterns. In addition, MESI retrieves accurate flows over a much broader linear range and is especially more accurate for large flow variations^2,3^. Yet, most laboratory and commercial laser speckle contrast imagers still rely on the simple exposure approach as its practical implementation is much simpler^4,5^. In the MESI system developed previously in our group^6^, the acquisition of multiple exposure relies on a fixed camera exposure associated to laser pulses varying in duration and intensity as proposed previously^7^. This implies the use of an acousto-optic modulator (AOM) associated to an arbitrary waveform generator (AWG) and the precise synchronization of the camera to the laser pulses. This approach has been used by several groups in the last decade to produce most of the multiple exposure speckle contrast in vivo datasets in small animals so far^2,3,6–10^. We investigate here a much simpler and cost-effective instrumental approach. Synthetic exposure images for different exposure times are obtained by the summation of images acquired at a short exposure time (e.g. a 10 ms synthetic exposure image is obtained by the summation of 10 images obtained at 1 ms). This approach has been proposed previously using high sensitivity, low noise SPAD detectors but at the expense of a strongly limited spatial resolution^11^. Further studies have used synthetic exposure with high frame rate CMOS sensors but no systematic comparison to the laser modulation method was performed as a validation^12,13^. The present study was designed to compare the synthetic exposure approach using a standard CMOS camera to the laser modulation approach in typical conditions associated with blood flow imaging in mice brain^10^. First, we have characterized the noise of the CMOS camera used in the study. Second, we have compared the contributions to the raw speckle data of the different components of noise (dark, readout, shot) in both the synthetic exposure and the modulated laser approaches for increasing exposure times. Third, as the experimental conditions strongly affect the relative contribution of noises, we have studied (i) how the intensity of the detected light affects the shot noise contribution for different exposure times, and (ii) how the noises relative contributions affect differently the speckle contrast data in low flow or high flow conditions. Finally, we have compared noise corrected speckle contrasts and subsequent decorrelation times derived from both approaches. The limitations of a standard CMOS camera with limited frame rate are discussed considering the limited temporal sampling of the speckle decorrelation and subsequent bias for decorrelation times quantification.

## Results

In dynamic speckle imaging, the local contrast is estimated as the ratio of the local standard deviation divided by the local mean of pixels intensities (see Eq. (1) in “Imaging set-up and phantom description”). The underlying concept is that for fast flows, the speckle patterns decorrelates quickly resulting in a low local contrast, while for static areas, the speckle patterns remain identical, and the local contrast is high. The basics of speckle contrast imaging and the imaging instrumental set-up and flow phantoms are described in Methods “Speckle contrast calculation and relative flow evaluation” and “Imaging set-up and phantom description”. For speckle contrast imaging of a given flow under a fixed illumination, variances in the pixel intensities arise not only from the dynamic speckle patterns fluctuation related to the flow but also from noises (dark, readout, shot noises). In synthetic exposure, the successive addition of frames results in adding the different variances contributions for each pixel. Definition of parameters and equations for noise contribution evaluations are described in the Methods “Noises evaluation and correction”. To correct for the contributions not related to flow, the contributions of noises should be estimated thoroughly. We first characterize the CMOS sensor noise in “Results”. Then we evaluate the standard deviations corresponding to the raw signal and noises contributions in both acquisition modes (“Estimation of signal standard deviation contributions in modulated laser and synthetic exposure modes”) and how it affects the speckle contrast calculations (“Contributions of the noises to the speckle contrast in modulated laser and synthetic exposure modes”). In “Relative contribution of the shot noise to the contrast as a function of illumination intensity” and “Relative contribution of shot noise to the contrast as a function of the measured flow” we investigate how the experimental conditions (detected intensity, velocity of the imaged flows) affect the relative contribution of noises to the signal. In “Comparison of flow quantification from data acquired using modulated laser or synthetic acquisition”, we compare both acquisition modes for relative flow measurements relative and absolute quantification.

### Experimental characterization of dark and readout noises for a CMOS sensor

**Figure 1:**
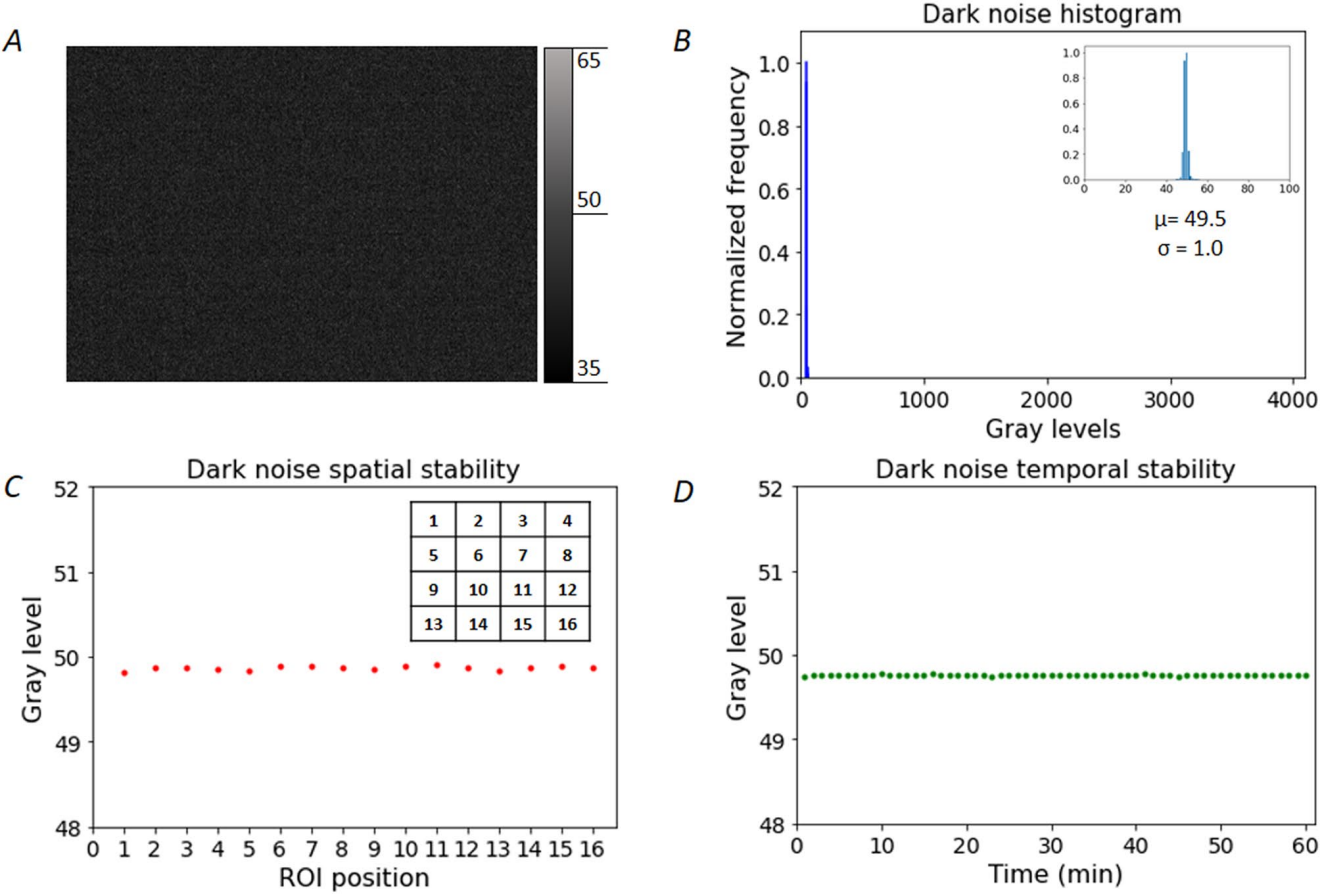
Camera dark and readout noise homogeneity and stability. **(A)** Representative dark image. **(B)** Corresponding histogram of grey levels. Insert is a zoom on non-zero histogram levels. **(C)** Spatial uniformity of noise, the sensor was divided to 16 regions of identical dimensions numbered from 1 to 16. **(D)** Temporal stability of noise evaluated as the mean grey level of the whole sensor.

Figure 1 shows the experimental characterization of the dark and readout noises of the Orca Flash 2.8 camera operated in simple exposure mode (e.g., each exposure time corresponds to the acquisition of a single independent image at this exposure). As can be seen, the dark and readout noises are low and homogenous for all pixels (Fig. 1A,B). In addition, the dark and readout noises are spatially homogeneous (Fig. 1C) and temporally invariant (Fig. 1D) making the subtraction of this contribution straightforward for this specific camera.

### Estimation of signal standard deviation contributions in modulated laser and synthetic exposure modes

**Figure 2:**
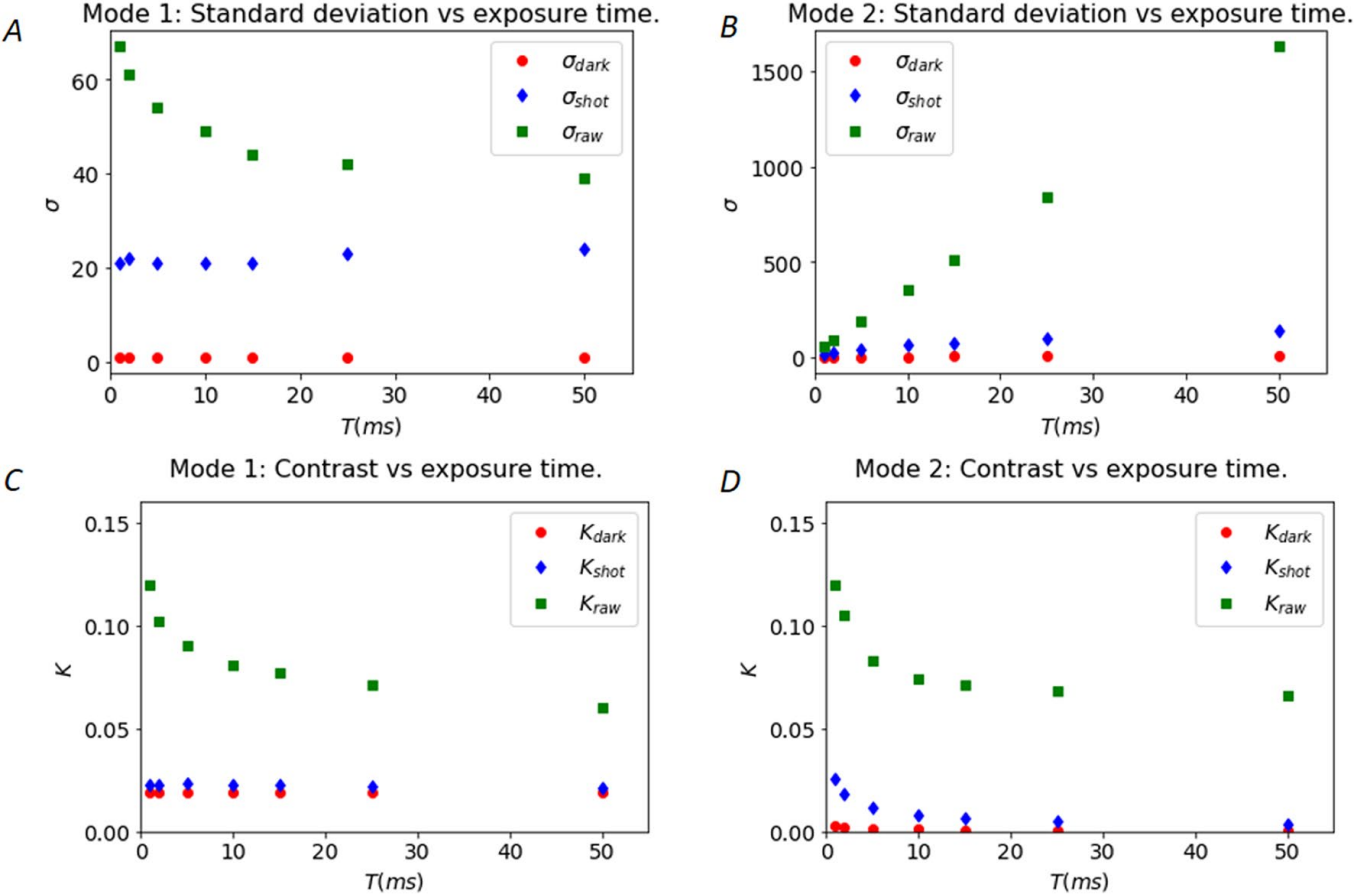
**(A)** Raw, dark and readout, and shot noise standard deviations in the modulated laser mode as a function of exposure time. **(B)** Same as **(A)** for the synthetic exposure mode. **(C)** Raw, dark and readout and shot noise speckle contrasts for the modulated laser mode. **(D)** Same as **(C)** for the synthetic exposure mode.

The standard deviation of the signal intensities is at the core of the speckle contrast calculation. The direct subtraction of noise to the signal images allows correction of the pixel intensities, but subtraction leads to an added variance. Uncorrelated variances components are additive. Therefore, the variance due to dark, readout and shot noises should be evaluated and subtracted from the raw variance to obtain a correct estimation of the speckle contrast related to the velocity of the moving red blood cells (see calculation methods and equations in the “Noises evaluation and correction” Methods section). Figure 2A,B shows the experimental evaluation of the raw, dark and readout noise, and shot noise standard deviation as a function of the exposure time in the modulated laser mode (mode 1) and in the synthetic exposure mode (mode 2). The standard deviation of dark, readout and shot noise remain constant for all exposures for the modulated laser mode whereas the shot noise related variance increases linearly with the number of frames (i.e. the exposure time) in the synthetic exposure mode (the standard deviation increases as the square root function of the exposure time as seen on Supplementary Fig. S1). In mode 1, the amount of detected light is maintained constant for all exposure times by adjusting the laser intensity with the duration and the amplitude of the pulses. Therefore, the shot noise variance and standard deviation remain constant. Contrarily, in mode 2, the computed detected signal adds with the number of frames resulting in a much higher dynamic for the synthetic image than what is allowed by the limited depth well of the pixels. In other words, if a pixel is already close to intensity saturation for a single image at 1 ms exposure time, adding 10 frames will not saturate the synthetic 10 ms image like a real single 10 ms exposure would. Regarding the dark and readout noises, as only one image is read for each exposure time in mode 1, the readout and dark variance and standard deviation are independent of the exposure time, provided that no varying ambient light is recorded, and no temperature changes occur. In mode 2, individual frames are added to create long exposure times, so the dark and readout variance should increase linearly with the exposure time and the standard deviation should increase following the square root of the exposure time. For the shot noise this assumption is met for all exposure times while for the dark noise the standard deviation shows a two terms dependency (sum of square root and a linear term) with the exposure time (see Supplementary Fig. S1). This unexpected linear contribution is predominant for longer exposure time and is likely due to a dark fixed pattern noise becoming significant for the summation of several frames. However, this does not significantly affect the value of the corrected contrast as for long exposure times, *σ*_*shot*_ is largely predominant as it is about one order of magnitude larger than that of *σ*_*dark*_.

### Contributions of the noises to the speckle contrast in modulated laser and synthetic exposure modes

The metric of interest for flow evaluation in speckle imaging is the local speckle contrast. Figure 2C,D show the raw speckle contrast as well as the contribution of the dark and shot noises contrasts for both acquisition modes as a function of exposure time (see “Noises evaluation and correction” for calculation methods). As expected**,** these contributions remain constant throughout the whole range of exposure times for the modulated laser approach. On the contrary, for synthetic exposures, the contributions to the speckle contrast of the shot and the dark noise contrasts decrease with the exposure time (i.e. the number of added frames). The standard deviation for the shot noise increases as the square root of the number of frames (see Supplementary Fig. S1), whereas the signal increases linearly with the number of frames leading to a theoretical decrease of the contribution of each noise to the speckle contrast as $\frac{1}{\sqrt{N.{I}_{one frame}}}$ (as shown on Supplementary Fig. S2) where *N* is the number of frames and *I*_*one frame*_ is the intensity level of one frame in electrons unit. The contributions to the contrast of the shot and dark noises contrasts are significantly lower in mode 2 for all exposures except the shortest ones.

### Relative contribution of the shot noise to the contrast as a function of illumination intensity

**Figure 3:**
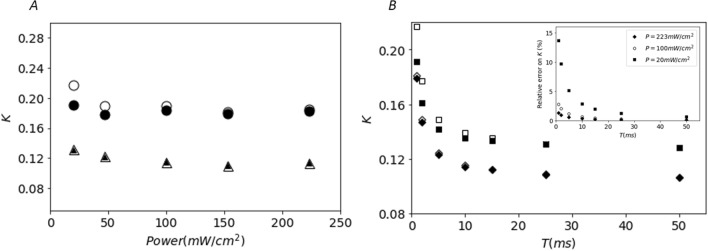
Effect of illumination level and exposure time on the shot noise contribution to the speckle contrast in synthetic exposure mode. (**A**) Effect of the detected light intensity; empty dots (raw contrast *K*_*raw*_) and filled dots (noise corrected contrast *K*_*corr*_) correspond to an exposure time of 1 ms; empty triangles (*K*_*raw*_) and filled triangles (*K*_*corr*_) correspond to an exposure time of 25 ms. (**B**) Effect of the exposure time; empty squares (*K*_*raw*_) and filled squares (*K*_*cor*r_) correspond to an illumination of 20 mW/cm^2^; empty diamonds (*K*_*raw*_) and filled diamonds (*K*_*corr*_) correspond to an illumination of 223 mW/cm^2^. Insert: Relative error due to the shot noise speckle contrast as a function of exposure time for different illumination levels; squares, dots and diamonds correspond respectively to illuminations of 20, 100 and 223 mW/cm^2^.

The relative contribution of the shot noise to the raw speckle contrast depends on the detected signal intensity. In mode 2, the shot noise contribution is determined by the level of detected signal at the lowest exposure time and the number of frames that are added to compute the speckle patterns for each exposure time. Figure 3 shows the influence of the noise correction for different levels of illumination of the sample, ranging from optimal use of the camera dynamic range (quasi saturation of the camera well depth) to sub-optimal level of signal (less than half of the pixels depth well are used). For optimal use of the camera dynamic, the contribution of the shot noise is almost negligible even for the shortest exposure time (Fig. 3A, illumination at 223 mW/cm^2^, *T* = 1 ms). When the signal level is sub-optimal, the relative contribution of the shot noise is significantly higher. This is likely to occur when speckle imaging is carried out over a large field of view as the laser power is distributed over a large surface (Fig. 3A, illumination at 20 mW/cm^2^, *T* = 1 ms). Figure 3B, shows that the relative contribution of shot noise to the contrast increases also when short exposure times are required to catch the contrast variations due to large flows. As a summary, the insert in Fig. 3B shows that the contribution of shot noise to the speckle contrast rises to 15% when speckle patterns are acquired under low illumination and for short exposure time. On the contrary, this contribution is limited to less than 2% for all exposure times when optimal illumination is obtained.

### Relative contribution of shot noise to the contrast as a function of the measured flow

**Figure 4:**
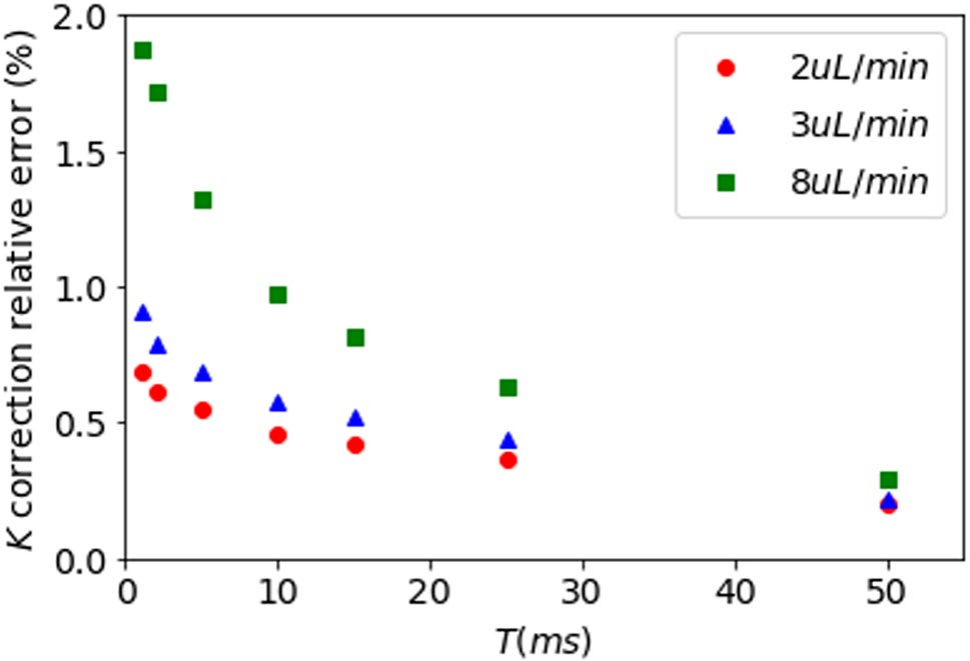
Relative error on contrast calculation due to noises contribution as a function of exposure time for different flows of intralipid-2% in a 300 µm diameter channel. Data have been acquired in synthetic exposure, with frames of 1 ms exposure time, and under optimal illumination using the full dynamic of the camera sensor.

The absolute contribution of the shot noise is obviously independent of the flow that is imaged. However, the relative contribution of shot noise to the speckle contrast depends on the value of the flow-related speckle contrast. In dynamic speckle imaging, high flows result in lower contrast whereas low flows lead to higher contrast values. Consequently, for the same level of illumination, the relative contribution of the shot noise to the total speckle contrast varies with the flow being imaged. Figure 4 shows that the relative contribution of noise is much higher for a flow of 8 µl min^−1^ compared to a flow of 2 µl min^−1^ for the whole range of exposure times considered, while as shown previously, the shot noise contribution decreases with the exposure time for all flows. Nevertheless, as data have been acquired under optimal illumination, it can be observed that the error remains below 2%.

### Comparison of flow quantification from data acquired using modulated laser or synthetic acquisition

**Figure 5:**
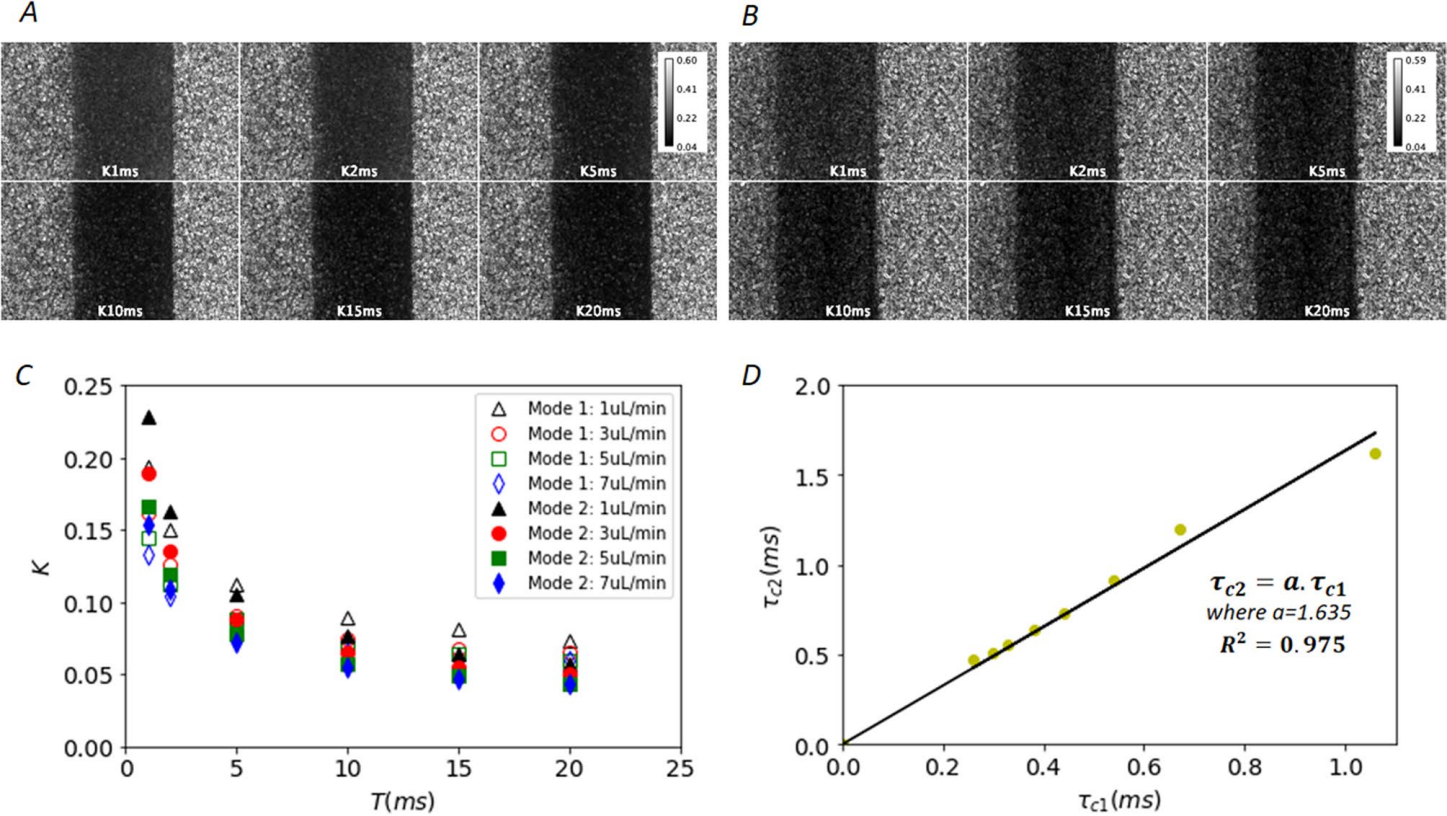
Comparison between mode 1 (laser modulated method) and mode 2 (synthetic exposure). **(A)** Speckle contrast images of intralipid-2% flowing at 3 µl/min in a 300 µm diameter channel for 6 exposure times (mode 1). **(B)** Same as **(A)** for mode 2. **(C)** Speckle contrast in both modes for intralipid-2% flowing at 1, 3, 5 and 7 µl min^–1^ in a 300 µm diameter channel versus exposure time (1, 2, 5, 10, 15 and 20 ms). Open symbols correspond to mode 1, filled symbols correspond to mode 2. **(D)** Decorrelation times for mode 2 data (${\tau}_{c2}$) as a function of those derived from mode 1 data (${\tau}_{c1}$) for all flows ranging from 1 to 8 µl min^−1^.

Data were acquired sequentially in both modes for controlled flows between 1 and 8 µl/min of 2%-intralipid in a 300 µm microchannel. Representative speckle contrast images for 6 different exposure times (1, 2, 5, 10, 15 and 20 ms) are shown for acquisition mode 1 and mode 2 on Fig. 5A,B respectively. Figure 5C shows representative noise corrected speckle contrasts in both modes for flows ranging from 1 to 8 µl min^−1^. Figure 5D shows the decorrelation times obtained from the synthetic exposure as a function of those obtained from the modulated laser acquisition. The decorrelation times for different flows are derived from a fit of Eq. (2) to the data (see “Speckle contrast calculation and relative flow evaluation”). For the phantom used, the decorrelation times measured in the modulated laser mode are within 0–1.2 ms, which is representative of what was observed in vivo in mice using a direct measurement of the intensity autocorrelation function g_2_(τ) (see Fig. 2D in Postnov et al. 2020^14^). Experimental data show a strong linear relationship between the decorrelation times obtained from both acquisition modes, yet the slope is not unity. The decorrelation times derived from mode 2 are significantly higher than those derived from mode 1. The reasons of this difference are not totally clear. Although it prevents absolute quantification of the decorrelation times, the measurement of relative flow changes in the considered range is possible using the synthetic exposure approach. This important point on the biased absolute decorrelation time quantification in mode 2 is further discussed in “Limitations of the synthetic exposure MESI using a standard CMOS camera”.

## Discussion

### Camera noises contributions to the speckle contrast

In the present study, we use a CMOS camera which was first issued by Hamamatsu in 2012. Its modest specifications in terms of noises and sensitivity compared to more recent sCMOS cameras do not hinder MESI acquisitions in the synthetic exposure mode. Obviously, higher spectral efficiency, and lower dark and readout noise would further decrease the contribution of noise to the speckle contrast, yet the main noise contribution to the speckle contrast arises from the shot noise, which is inherently independent of the camera. However, specifications of common CMOS cameras, even from established manufacturers should be considered cautiously for speckle imaging in the synthetic exposure mode. Indeed, in CMOS cameras, the charge to voltage conversion is independent for each pixel and each column has its own amplifier and analog-to-digital converter, in addition to possible pixel-to-pixel variation in quantum efficiency. The “raw” output from the CMOS image sensor includes pixel-to-pixel variability in the read noise, electronic gain, offset and dark current, which are corrected by the manufacturer in high performance sCMOS cameras^15^ but not in regular CMOS cameras. Consequently, standard CMOS usually have moderate to strong inhomogeneous spatial noise (quantified as Dark Signal Non-Uniformity, DSNU), spatial patterns of noise arising from the architecture of the electronics, which should be considered cautiously if systematic noise correction is carried out. Although laser speckle imaging set ups have been implemented with moderate performance cameras like webcams, it is unlikely that such approach would allow unbiased multiple exposure imaging in the synthetic exposure mode^16^ as successive frames summation would make the uncorrected noise pattern more visible and ultimately bias locally the speckle contrast calculation.

### Illumination level and uniformity

A general guideline for laser speckle imaging is that the illumination of the imaged tissues should be set to maximize the use of the camera dynamics and to minimize the shot noise contribution. The shot noise contribution to the contrast is maximal when the illumination level is sub-optimal (Fig. 3). In the synthetic exposure mode this can be true for low exposure times while longer exposure times benefit from the summation of multiple frames. Similarly, in the study by Valdes et al.^17^, the shot noise contribution overwhelms the flow-related speckle contrast for tissues areas with a low signal to noise ratio. The contribution of the shot noise under low illumination could be problematic in the case of clinical application of speckle contrast imaging as practical and safety constraints impose the use of low power laser beams expanded over wide field of view thus limiting the amount of signal^18^. In these conditions, the synthetic exposure mode could be particularly interesting compared to the modulated laser mode. It would allow to strongly decrease the shot noise contribution to the speckle contrast for long exposure times requested to probe small flow changes. The interpixel variability in sensitivity and electronic conversion chain is quantified as the photon response non uniformity (PRNU) for CMOS cameras. In recent sCMOS cameras, it is carefully corrected^15^ similarly to DSNU avoiding unwanted spatial patterns that could bias the speckle images in the synthetic exposure mode. Another issue, not specific to synthetic acquisition, is the importance of homogeneous illumination for speckle contrast calculation, as inhomogeneous illumination leads to biased flow maps due to inhomogeneous shot noise contributions within the field of view^19,20^. Although calculating speckle contrast in the temporal dimension solves this issue^21,22^, in the current implementation of the synthetic exposure mode, the speckle contrast is calculated using spatial kernels and the non-uniformity of field illumination should be minimized or corrected as differences add with the sum of frames.

### Relative contribution of noises as a function of the experimental conditions

An important finding from this study is that the relative contribution of noise to the speckle contrast depends on several intermingled experimental parameters. As mentioned above, the amount of available light (laser power per mm^2^ on the sample) is essential, as detected signals far below camera saturation increase the relative contribution of the shot noise to the speckle contrast. Furthermore, the flow that is imaged is an important parameter. Slow flows observed in small arterioles, capillaries and the parenchyma result in slow decorrelation of the speckles and high speckle contrast values, thus minimizing the relative contribution of the noise-related contrasts. On the contrary, imaging fast flows as those observed in arteries or large arterioles is much less favorable since rapid speckle decorrelation leads to low speckle contrast values. In addition, speckle contrast imaging of fast flows requires short exposure times, which could lead to sub optimal illumination. To avoid such discrepancies, we advise a systematic correction of the shot noise related contrast from all speckle contrast data.

### Limitations of the synthetic exposure MESI using a standard CMOS camera

The limited frame rate and subsequent non negligeable interframe delay is a strong limitation of the synthetic exposure approach using a standard CMOS camera. Indeed, interframe delays should be kept at the shortest values to provide accurate temporal sampling of the decorrelating speckle patterns in respect of the Nyquist -Shannon theorem. The modulated laser approach allows to sample properly the course of the speckle patterns decorrelation as data for each exposure are contiguous (with a null interframe delay). Similarly, previous implementations of synthetic exposure MESI have used (i) single photon avalanche detectors (SPAD) with excellent speed and sensitivity but limited number of pixels and spatial resolutions^11^ or (ii) custom CMOS hardware sensors coupled to FPGA allowing frame rates of 15kHz^12^ or 1 kHz ^13^ respectively with negligeable interframe delays (a few µs) in regards of the decorrelation times. For SPAD, synthetic summation of frames is possible after noise corrections, yet the spatial resolution and the number of "pixels" is coarse. For CMOS detectors contiguous data acquisition requires dedicated electronics and complicated hardware including FPGA treatment and analysis. Our implementation is based on a CMOS camera with a 46 Hz frame rates at full frame up to 714 Hz for a region of interest limited to 50 × 50 pixels with respective interframes delays of 21 ms and 0.4 ms (see Supplementary Fig. S3). These interframe delays result in the violation of the Nyquist-Shannon theorem when decorrelation times are short and consequently lead to incomplete capture of the temporal pattern of the speckle decorrelation. Still, our experimental data suggest that some of the decorrelation is captured as the comparison with AOM acquired data (with null interframe) demonstrates a linear relationship between the decorrelation times derived from both methods for the same flows. Although the absolute quantification of decorrelation times is out of reach, the implementation of synthetic exposure MESI with a standard CMOS allows to measure relative flow speed changes over a range of decorrelation times that are representative of in vivo biological data obtained using direct autocorrelation measurement (see Fig. 2D in Postnov et al.^14^).

### Temporal resolution of synthetic exposure mode

The overall temporal resolution of the blood flow measurements is the total duration of the time window of the data required to derive one value of the decorrelation time. In the modulated laser mode, it depends on the number of exposure times used and on the duration of the longest exposure time that sets the constant exposure of the camera. In a standard MESI implementation, 6 exposure times are used ranging from 1 ms up to 20 ms^9,10^. The total duration of a dataset required for the evaluation of one decorrelation time and the corresponding blood flow index is about 120 ms (6 × 20 ms). In the synthetic exposure mode, the temporal resolution is limited by the maximum frame rate of the camera. In our implementation, the frame rate ranges from 46fps (full frame sensor) up to 714fps (50 × 50 pixel ROI) leading to a temporal resolution of 430 ms and 28 ms respectively for 20 images at 1 ms exposure datasets. A resolution of 430 ms is a coarse temporal resolution, yet within the order of magnitude of the characteristic time of physiological blood flow changes. For instance, local increase of cerebral blood flow occurs typically within 0.5–1 s post sensory stimulation^23–25^.

### Guidelines for synthetic exposure using a standard CMOS camera

The synthetic exposure scheme with an off-the-shelf CMOS camera benefits from a straightforward instrumental implementation. An essential feature, regarding the camera choice, is a low, stable, and uniform noise as well as a high sensitivity and a homogenous sensor response. Also, an optimal, homogeneous, and stable illumination is requested to ensure that the full dynamic of the camera is used and to reduce the contribution of shot noise even under unfavorable conditions often met for blood flows analysis in living tissues (short exposure times, fast flows). Ensuring constant illumination over the whole exposure times acquisition is much easier to achieve in the synthetic exposure mode compared to the AOM time-intensity modulation. Indeed, for AOM modulation, the intensity for each pulse has to be pre-calibrated in advance on a reference object and is hardware coded using an arbitrary wavelength generator to modulate the intensities of each laser pulses. This pre-calibration step leads to differences in detected illumination intensities when imaging real biological tissues as the exposed object has a priori unknown heterogeneous optical properties and shapes. The timing and readout speed of the camera is also an essential parameter. As discussed in “Limitations of the synthetic exposure MESI using a standard CMOS camera”, the interframe delay of the camera should be kept to a minimum, ideally null, and it should be at least twice shorter than the measured decorrelation times to ensure proper temporal sampling of the speckle decorrelation. In our implementation, the interframe delay and overall temporal resolution of the multiple exposure data are limited by the frame rate of the camera. Selecting smaller ROIs on the sensor allowed to increase the frame rate and consequently decreased the interframe down to 0.4 ms. For up-to-date CMOS cameras, an ROI of 300 × 300 pixels result in frame rates up to 900 Hz at 1 ms exposure time. Such high frame rates correspond to interframe delay of 0.1 ms and would fulfill the Shannon-Nyquist theorem for the temporal sampling of most speckle pattern decorrelations for blood flow in vivo. This would allow to get closer to absolute quantification of the decorrelation times which could not be obtained with the camera used in the present study.

## Methods

### Speckle contrast calculation and relative flow evaluation

For all conditions, contrast images were computed using the following equation:

1 $$K=\frac{\upsigma }{<I>}$$

where *σ* is the standard deviation of the intensities in the acquired image, and < *I* > is the average intensity in the same image. All calculations were done on intensity images in electrons units. A sliding spatial window of 5 × 5 pixels is used for the calculation of the local contrast over the whole image. The assumption behind the speckle contrast imaging of biological flows is that the flow is inversely proportional to the decorrelation time *τ*_*c*_ of the scatterers. Using multiple exposure contrast images, a model was proposed to derive *τ*_*c*_ while considering the contribution of static scatterers^2^. Equation (2) is fitted to multiple exposure speckle contrast data as a function of exposure time. A non-linear least square approach implemented using Levenberg–Marquardt algorithm was used.

2 $$K\left(T,\tau c\right)={\left\{\beta {\rho }^{2} \frac{{e}^{-2x}-1+2x}{{2x}^{2}}+4\beta \rho \left(1-\rho \right) \frac{{e}^{-x}-1+x}{{x}^{2}}+ {\upsilon }_{noise} \right\}}^{1/2}$$

where $T$ is the camera exposure time, $\beta$ is a unitless constant that accounts for spatial averaging of the spekle grains and instrumental parameters, $\rho$ is defined as the ratio of the intensity contribution of mobile scatterers over the total intensity due to mobile and static scatterers, *x* is the ratio of *T* over ${\tau }_{c}$, and *v*_*noise*_ accounts for experimental noises contributions.

The decorrelation time *τ*_*c*_ is derived from the fit and the relative blood flow index (BFI) is defined as

3 $$BFI= \frac{1}{{\tau }_{c}}$$

### Imaging set-up and phantom description

**Figure 6:**
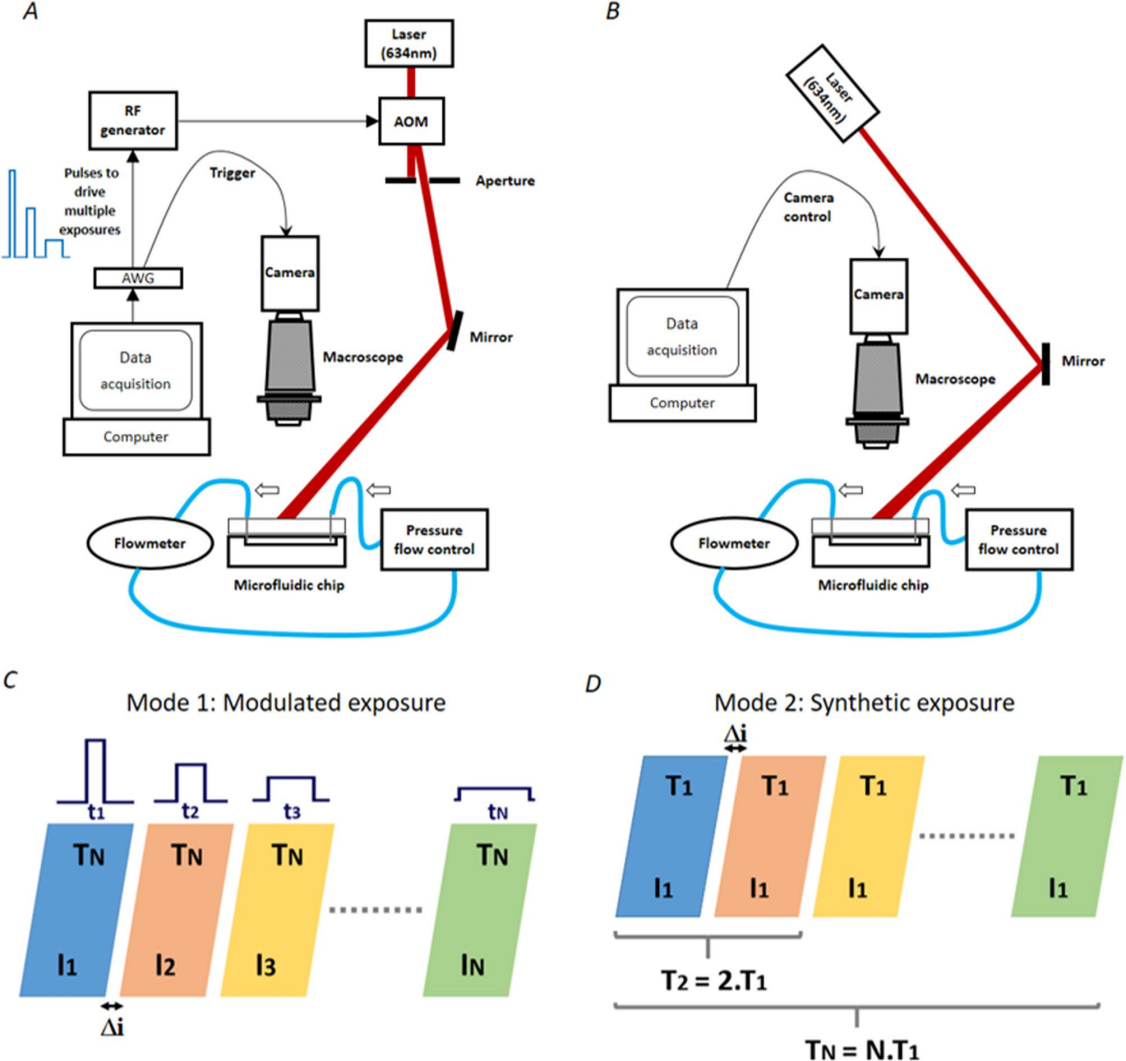
Imaging set up and acquisition modes **(A)** Modulated laser mode set-up (mode 1). *AWG* arbitrary waveform generator, *AOM* acousto-optic modulator. **(B)** Synthetic exposure mode set-up (mode 2). **(C)** Modulated laser acquisition scheme. *N* is the number of different exposures at which the speckle patterns are recorded. The camera exposure time *T* is fixed and the laser is modulated in duration *t* and intensity *I*. **(D)** Synthetic exposure acquisition scheme. The laser is operated in continuous mode, its intensity *I* is fixed, the camera exposure time is fixed and set to the shortest exposure time *T*_*1*_. For both modes, Δi is the camera interframe time.

The imaging set-up is showed in Fig. 6. A 634 nm laser diode with a maximum output power of 419 mW is used. Images were acquired through a stereomicroscope (Leica MZ16) with a genI sCMOS camera (Orca Flash 2.8, Hamamatsu, Japan). It has a 42% spectral sensitivity at 634 nm, a typical read noise of 3e− and a dark noise of 1e−. It is composed of 1920 × 1440 square pixels. The microfluidic channels fabrication process and flow control set-up were described in details previously^6^. Images were acquired for various flows in microfluidic channels made of clear PDMS with a rectangular section of 300 µm widths, 75 µm of height and 1 cm of length. The channel is sealed with a lid of clear PDMS, with no static scatterers added. On the basis of previous studies^23,25^, we used 2%-intralipid as the flowing media since it is easily available in large volumes, non-toxic and its reduced optical scattering coefficient is close to that of flowing blood^26,27^. Flows from 1 µl min^−1^ up to 8 µl min^−1^ are controlled using a pressure controller with inline flow feedback (Fluigent, France). In our set-up, these flows correspond to speeds ranging between 0.7 and 5.9 mm s^−1^ representative of blood speeds in capillaries and arterioles.

### Multiple exposure acquisition modes

Multiple exposure speckle data were obtained for the following exposure times: 1, 2, 5, 10, 15, and 20 ms. In a first approach, called laser modulation method (Fig. 6C, mode 1), the acquisition of multiple exposure relies on a camera exposure fixed at 50 ms synchronized to laser pulses varying in duration and intensity using an acousto-optic modulator. The width of each pulse is set to the desired exposure duration, while its amplitude is set so that the pulses energies are equalized. The shortest exposure time requires the maximum amplitude, whereas the longest exposure is associated with the lowest amplitude. Contrast images are then calculated for all exposure times using Eq. (1) and *τ*_*c*_ is derived from a fit of the contrast data to Eq. (2). In a second approach, called synthetic exposure (Fig. 6D, mode 2), contrast images are generated using synthetic exposures as follows: the camera exposure time is set to 1 ms and the laser diode power is adjusted to ensure that the maximum dynamic of the sensor is used. 20 independent frames at 1 ms are acquired and contrast images for *T*: 1, 2, 5, 10, 15, and 20 ms are computed as the sum of the corresponding number of 1 ms-frames. For each exposure time, a single summed-up image was used to evaluate the speckle contrast. When used in full frame, the camera interframe duration Δi is set to 21 ms by the readout speed electronics. Uncorrelated short exposure frames (1 ms) are summed to create longer exposure times. Data at shorter interframe delays have been obtained at the expense of a reduction of image dimension. (Supplementary Fig. S3).

### Noises evaluation and correction

Speckle contrast relies on the statistical properties of the local signal intensity and is calculated using Eq. (1) and a 5 × 5 spatial kernel. Following this method, local uncorrected mean intensity *I*_*raw*_ and standard deviation $\sigma$_*raw*_ can be computed from the raw data leading to the raw speckle contrast

4 $${K}_{raw}=\frac{{\sigma }_{raw}}{\langle {I}_{raw}\rangle }$$

However, there are several noises contributions arising from the detector including dark noise, readout noise and shot noise. Here, we evaluate these contributions to obtain the noise-corrected speckle contrast *K*_*corr*_. First, a stack of dark images at 1 ms exposure time is acquired with the laser source off to account for dark and readout noises simultaneously. From these, we evaluate the intensity *I*_*dark*_, variance and standard deviation $\sigma$_*dark*_ and resulting speckle contrast *K*_*dark*_ for each synthetic exposure time.

5 $${K}_{dark}=\frac{{\sigma }_{dark}}{\langle {I}_{dark}\rangle }$$

$\sigma$_*raw*_ and *I*_*raw*_ are computed from raw speckle images acquired on a sample (e.g. a channel filled with 2%-intralipid pumped at a precisely known flow). Dark noise intensity is subtracted from each raw image leading to *I*_*corr*_ which corresponds to intensities corrected from the dark and readout intensities.

6 $${I}_{corr}={I}_{raw}-\langle {I}_{dark}\rangle$$

From this resulting image we can compute the variance ${\sigma }_{corr}^{2}.$

However, the contribution of dark noise fluctuations ${\sigma }_{dark}^{2}$ should be subtracted from the variance ${\sigma }_{corr}^{2}$.

The contribution of the shot noise obeys to Poisson’s statistics with a variance ${\sigma }_{shot}^{2}$ proportional to the mean resulting in a shot noise related contrast contribution calculated as:

7 $${K}_{shot}=\frac{1}{\sqrt{{\upgamma I}_{corr}}}$$

where *γ* is the ratio of full well capacity of the CMOS sensor to its analog-to-digital conversion bits.

Finally, the speckle contrast corrected for both the shot noise and dark-readout noise is given as^17^

8 $${K}_{corr}=\sqrt{\frac{{\sigma }_{corr}^{2}-{\sigma }_{shot}^{2}-{\sigma }_{dark}^{2}}{{\langle {I}_{corr}\rangle}^{2}}}$$

## Supplementary Information

Supplementary Figures.

## Data Availability

The datasets generated during the current study are available from the corresponding author on reasonable request.
