# Supplementary material for: Synthetic exposure with a CMOS camera for multiple exposure speckle imaging of blood flow
Source: Sci Rep. 2022 Mar 18;12:4708. doi: 10.1038/s41598-022-08647-6 (PMC8933569; doi:10.1038/s41598-022-08647-6)

**Synthetic exposure with a CMOS camera for multiple exposure speckle imaging of blood flow**

[1]Chammas M, [*,1]Pain, F

[1]*Université Paris-Saclay, Institut d'Optique Graduate School, CNRS, Laboratoire Charles Fabry, 91127, Palaiseau, France.*

**corresponding author: frederic.pain@universite-paris-saclay.fr*

## Supplementary materials

**Supplementary Figure S1.** Shot and dark noises standard deviations as a function of the exposure time (i.e. the number of summed images) in the synthetic exposure mode.

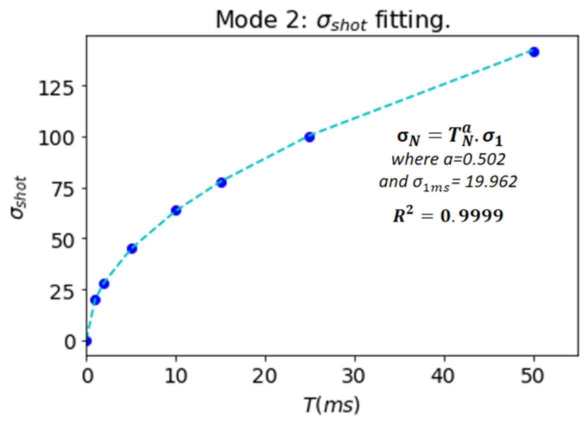


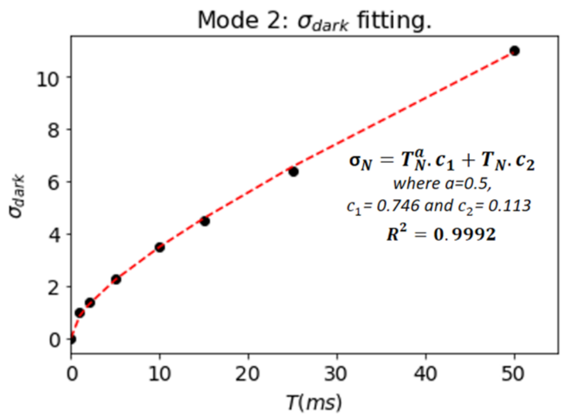


**Supplementary Figure S2.** Shot noise contrast $K_{shot}$ as a function of exposure time (i.e. the number of summed images) in the synthetic exposure mode.

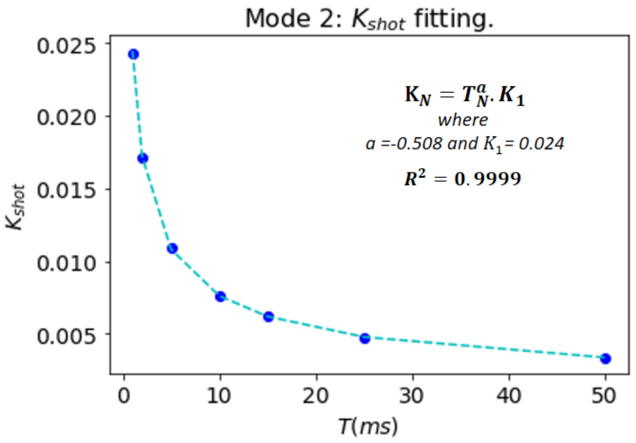

**Supplementary Figure S3.** Effect of frame rates and subsequent interframes durations on the evaluation of the speckle contrast $K_{corr}$ in the synthetic exposure mode.

Noise corrected speckle contrast $K_{\mathrm{corr}}$ as a function of exposure times for different flows (intralipid-2% in a 300 µm channel) and increasing frame rates. Full frame acquisition corresponds to 46fps and interframe $\Delta i = 21$ms. 500x500 pixels region of interest corresponds to acquisition at 122fps and $\Delta i = 7.2$ms. 50x50 pixels region of interest corresponds to acquisition at 714fps and $\Delta i = 0.4$ms. Red symbols correspond to Brownian motion. Blue symbols correspond to 1 $\mu l.min^{-1}$ flow and black symbols to 3 $\mu l.min^{-1}$ flow.

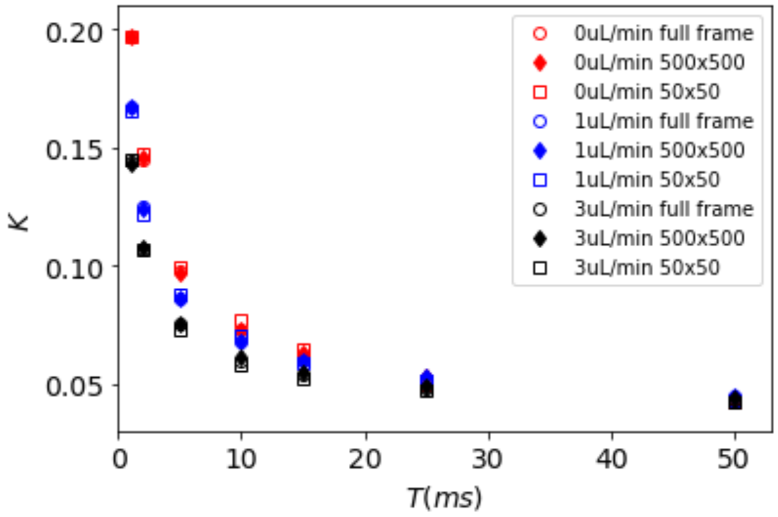

Supplement: Supplementary file 1 — Supplementary Figures. [file 41598_2022_8647_MOESM1_ESM.pdf]
